# The network neuropsychology of neighborhood deprivation in juvenile myoclonic epilepsy

**DOI:** 10.1038/s41598-026-46473-2

**Published:** 2026-04-16

**Authors:** Camille Garcia-Ramos, Bruce P. Hermann, Vivek Prabhakaran, Veena A. Nair, Dace N. Almane, Anusha Adluru, Nagesh Adluru, Jana E. Jones, Aaron F. Struck

**Affiliations:** 1 Department of Neurology, University of Wisconsin-Madison, Madison, WI 53726 USA; 2 Department of Neurology, Washington University, St Louis, MO 63110 USA; 3 Department of Radiology, University of Wisconsin-Madison, Madison, WI 53726 USA; 4 Waisman Center, University of Wisconsin Madison, Madison, WI USA

**Keywords:** Neuropsychology, Graph theory, Juvenile Myoclonic Epilepsy, Networks, Neurology, Neuroscience, Psychology, Psychology

## Abstract

Using network analytics, we sought to determine whether neighborhood disadvantage was associated with disruptions to the intrinsic cognitive networks of persons with Juvenile Myoclonic Epilepsy (JME). 62 participants with JME were categorized into high (*n* = 18) and low (*n* = 44) disadvantaged groups using the Area Deprivation Index and compared to 44 controls as to the network properties of their cognitive networks. The networks were interrogated using test metrics from a comprehensive neuropsychological test battery that assessed intellectual ability, language, visouperception/construction, learning and memory, executive function, and speed-dependent abilities. Network analyses demonstrated graded increases in overall connectivity and association strength across groups (controls < low-disadvantage JME< high-disadvantage JME), with the high-disadvantage JME group showing the greatest number of positive correlations and strongest inter-test associations. Community structure differed across groups, with reduced modularity and less differentiated cognitive networks in JME, particularly in the high-disadvantage group, suggesting over-integration and reduced network segregation. Regression analyses identified antiseizure medication load as a significant predictor of global efficiency in low-disadvantage JME only, with no significant clinical seizure predictors observed in the high-disadvantage JME group. The results indicate that structural (neighborhood) disadvantage is associated with detectable adverse effects on the underlying cognitive networks of persons with JME. While the cognitive status of JME has long been known to be adversely affected, the results reported here demonstrate that fundamental shifts in underlying cognitive network properties are associated with disadvantage, characterized by an abnormally highly integrated and less modular (differentiated) network structure.

**Supplementary Information:**

The online version contains supplementary material available at 10.1038/s41598-026-46473-2.

## Introduction

An extensive global literature has demonstrated that the adequacy of cognition in youth and adults with epilepsy can be adversely impacted by diverse clinical seizure features (e.g., epilepsy syndrome, age of onset, duration), antiseizure medication type and number, somatic and psychiatric comorbidities, and abnormalities in brain structure, connectivity, and metabolism^1–4^.

More recently, an emerging literature has reported that the adequacy of neuropsychological, behavioral and quality of life status of youth and adults with epilepsy is inversely associated with structural as well as personal markers of disadvantage. Several reports have used neighborhood metrics of disadvantage, such as the Area Deprivation Index (ADI)^5^, demonstrating that greater disadvantage is associated with more impaired cognitive status in adults with chronic temporal lobe epilepsy^6^ and youth with new and recent onset epilepsies^7^ including a slowed rate of cognitive-intellectual development over a two-year course^8^. Personal metrics of disadvantage (e.g., family income, parental marital status, parental employment) have also been shown to be associated with poorer academic achievement, neuropsychological status, and behavioral adjustment both at the time of diagnosis and out to three years following diagnosis among youth with new onset epilepsies^9–11^.

In this context, the conceptual focus of the neuropsychology of epilepsy has evolved over time. Initially, efforts centered on the administration of tests of general mental ability to characterize the impact of epilepsy on cognition^12,13^. With the advent of electroclinical characterization of seizures and syndromes^14,15^, and the development of formal internationally informed taxonomies of epilepsy^16^, the field shifted to identifying prototypical syndrome-specific cognitive abnormalities within a structure–function framework^17^. Most recently, the field has moved toward a network neuropsychology perspective^18–21^, aligning with the now-developed conceptualization of epilepsy as a disorder of disrupted local and remote brain networks^22^. The relationship of cognitive performance to underlying neurobiological networks and their differences compared to control populations has been key to these efforts, the networks reflected by diffusion, resting state or task-activated connectivity approaches^4^.

But it is important to remember that the cognitive measures administered in the context of a comprehensive test battery show significant relationships among themselves. These relationships have been interrogated by network science analytics^23^ and findings have revealed discernible anomalies in the underlying cognitive networks of persons with epilepsy^24^. Furthermore, the degree of network abnormality has been found to covary with the degree of neuropsychological abnormality as reflected in their relationship with so-called cognitive phenotypes that range from intact to focal to generalized impairment^25^. The question that arises here is whether there is a similar cognitive network signal associated with *disadvantage*. That is, can cognitive abnormalities be found to be associated with disadvantage at the “network neuropsychology” level^23^?

To address that question, here we examined the network characteristics of youth with a common form of generalized epilepsy—Juvenile Myoclonic Epilepsy. We have previously examined the impact of social determinants of health on the general cognitive status of JME^26^, but now investigate the network properties of this relationship.

## Methods

### Participants

Participants for this investigation came from the Juvenile Myoclonic Epilepsy Connectome Project (JMECP)^27^. The JMECP is a controlled cohort study with rolling recruitment and planned is prospective (2-year) reassessment. All available successfully recruited individuals served as participants. Cognitive data missingness was minimal (< 1%) and equivalent between groups. Eligibility for the JME group required participants to be 12–25 years old, fluent in English, and to have a confirmed diagnosis of JME based on at least two of the following: (1) clinical history or direct observation of early-morning myoclonic jerks; (2) clinical history or direct observation of generalized tonic–clonic seizures; and (3) EEG evidence of 3.5–5 Hz generalized spike-wave or polyspike-wave discharges. Exclusion criteria were: inability to provide informed consent; semiologic or EEG findings indicating potential focal epilepsy; any MRI-detectable lesion other than nonspecific white matter findings on a 3T epilepsy-protocol scan (including high-resolution axial T1-weighted MPRAGE and CUBE T2-weighted sequences); or seizures attributed to an active infectious process.

Control participants were excluded if they met any of the following conditions: (1) a prior history of an initial precipitating neurological event (such as simple or complex febrile seizures, cerebral infections, or perinatal stroke); (2) any documented seizure or seizure-like spell; (3) a diagnosed neurological disorder; (4) a history of loss of consciousness lasting longer than five minutes; or (5) a diagnosis of autism spectrum disorder.

Participants completed an extensive neuropsychological assessment covering intellectual functioning, language, visuoperceptual skills, learning and memory, executive abilities, and processing speed. All study procedures adhered to applicable regulations, received approval from the Institutional Review Board of the University of Wisconsin School of Medicine and Public Health (approval date: 2/3/2020), and were conducted in accordance with the Declaration of Helsinki. Written informed consent or assent was obtained from parents and children on the day of participation.

### Area deprivation index

The ADI is a factor-based index incorporating 17 US Census poverty, education, housing, and employment indicators to characterize neighborhood disadvantage in specific census-based regions. Socioeconomic disadvantage based on neighborhood risk through a ZIP code-linked ADI has the advantage of not requiring a lengthy and intrusive discussion with patients and families. The ADI from the 2015 American Community Survey (ACS) used here is a validated and widely used measure of neighborhood-level disadvantage^5^.

The home addresses of the epilepsy and control participants were entered into the University of Wisconsin’s Neighborhood Atlas (www.neighborhoodatlas.medicine.wisc.edu/*)*, which produces ADI metrics in terms of state/regional and national rankings. The participants were stratified into ADI quintiles, with those falling within the lowest two quintiles designated as the low-disadvantage (advantaged) subgroup and those in the highest two quintiles assigned to the high-disadvantage (disadvantaged) subgroup. Of the 62 individuals with JME, 44 were classified with low-disadvantage scores and 18 with high-disadvantage scores.

### Clinical and demographic data

Information on demographics, medical history, neuropsychological performance, neurological examination findings, and quality of life was collected using electronic forms consistent with NIH Common Data Elements guidelines. These data were compiled from chart reviews, questionnaires completed by participants, and a structured interview that captured detailed characteristics of seizure onset, types, and frequency.

### Cognitive data

Consistent with our earlier work^25^, the neuropsychological assessment consisted of tests pertinent to JME. Many of these measures were selected from the NINDS Epilepsy Common Data Elements, along with additional tasks from the NIH Toolbox Cognitive Battery. Table 1 shows the cognitive tests administered (1st column), notation (2nd column), cognitive domain assessed (3rd column), and specific ability represented (4th column). In the Results, we will discuss the averages for each group (5th -7th columns) and statistical comparison across groups (8th column).

### Graph theory measures and analysis

For each group, we generated symmetric matrices representing 15 standardized neuropsychological measures (treated as nodes). Weighted, symmetric adjacency matrices were then constructed by computing Pearson correlation coefficients for every pair of tests, adjusting for age. Because our goal was to examine the pattern of positive associations among tests without the interpretive complications introduced by negative correlations, only positive coefficients (excluding self-correlations) were retained. To accomplish this, matrices were thresholded to remove negative values and diagonal elements using the *threshold_absolute* function (threshold set to 0) from the MATLAB-based Brain Connectivity Toolbox (BCT), which was also used to compute the graph-theoretic (GT) indices.

Between-group differences were evaluated using a bootstrapping procedure in which each group was resampled with replacement 250 times. To model the contribution of random network structure to each GT metric, we also generated 250 random graphs per group, using the *randmio_und_connected* function of the BCT with 100 iterations, which matches node count and degree distribution, enabling formal testing against the null model. Multiple comparisons were controlled using the Bonferroni correction. GT metrics were derived from each resampled matrix, and group-level means were used for subsequent analyses.

We examined average global efficiency (GE) and clustering coefficient (CC), reflecting network integration and segregation, respectively, along with their normalized counterparts. High GE indicates a network in which information can travel efficiently due to the presence of numerous alternative routes between node pairs^28^. The CC quantifies segregation by calculating the proportion of actual connections among a node’s nearest neighbors relative to how many could exist theoretically^29^. In the context of cognitive networks, a high GE network suggests that cognitive abilities are well integrated, meaning that: (1) information (or variance) is shared efficiently across domains, (2) performance in one domain is strongly and indirectly related to others; (3) the cognitive system behaves more like a coordinated whole rather than isolated modules. Regarding CC, it reflects the extent to which related cognitive tests form tightly interconnected groups; reductions may indicate disrupted domain structure, whereas increases may reflect more rigid or over-coupled processing within domains. To minimize the influence of network size or other structural characteristics, both GE and CC were normalized by dividing each empirical value by the corresponding metric computed from the 250 random graphs in each group.

We also evaluated the community organization of each network, including modularity and the modularity index *Q*, which are jointly derived using the *community_louvain* algorithm. Community detection identifies sets of nodes that cluster together and likely participate in related functions, indicating network specialization. To enhance the interpretability of community assignment, the BCT function *grid_communities* was applied to the averaged matrices so that nodes were rearranged according to their module membership. Because the modularity algorithm yields probabilistic outcomes^30^, modularity was estimated 1,000 times for each group, and the most frequently occurring module configuration was selected. To reinforce the reliability of node assignments, we used a custom script to calculate, for each node, the proportion of times it was assigned to each module and then allocated the node to the module with the highest probability. The resulting community structures were visualized in two dimensions using the ForceAtlas2 layout implemented in the Gephi 0.10 software (https://gephi.org).

We further assessed graph hubs using betweenness centrality (BC), which identifies nodes that lie along the shortest paths connecting other node pairs and thus contribute disproportionately to efficient information flow^31^. Nodes with BC values exceeding the group mean by at least one standard deviation were classified as hubs. Finally, overall graph strength was computed to determine whether the groups differed in the general magnitude of their test-to-test correlations.

### Deriving subject-specific GT measures

To generate participant-specific GT metrics for subsequent correlations with demographic and clinical variables, we used the Add-One-Patient (AOP) procedure described by Saggar et al. (2015). In this approach, each JME participant is individually incorporated into the control group before computing the correlation matrix. The resulting matrix is then compared with the matrix from the control group alone, and the difference between the two yields a subject-level matrix representing that individual’s contribution to the network. This procedure was carried out separately for all JME participants (44 in the high-disadvantage group and 18 in the low-disadvantage group), after which GT measures were computed from each derived matrix. For the control group, subject-specific estimates were obtained using the complementary “leave-one-out” strategy^32^. To examine the clinical relevance of discrete GT metrics, a regression analysis was performed between subject-specific GT measures (GE, average CC, Q, and average BC) and epilepsy onset (in months), ADI scores, epilepsy duration, and number of antiseizure medications.

## Results

### Participants characteristics

Sociodemographic and clinical characteristics are presented in Table 2. The groups did not differ in age or sex (*p* > 0.5). ANOVA identified a significant group difference in mothers’ education (F_2,102_ = 6.613, *p* = 0.002), which is expected given its role as a socioeconomic status indicator. In addition, both JME groups were compared in terms of total months since most recent seizure (F_1,59_ = 1.928, *p* = 0.17), age of onset (F_1,33_ = 0.233, *p* = 0.63), and duration of disorder (F_1,55_ = 0.064, *p* = 0.801), and none showed significant differences between groups. All test scores were standardized and adjusted for age. The cognitive test scores used to construct the graphs were compared across the three groups using a MANOVA (see Supplemental File I). Significant group differences emerged in 13 of the 15 tests, with controls scoring highest, followed by the low-disadvantage group, and the high-disadvantage group scoring lowest/worst.

**Table 1:** Cognitive Assessment.

Test	Notation	Cognitive Domain	Specific ability	Unrelated controls (*n* = 44)	Low- disadvantage JME (*n* = 44)	High-disadvantage JME (*n* = 18)	Statistics
WASI Vocabulary	IQVOCS*	General ability-verbal	Word knowledge	11.93 $\:\pm\:\:$2.5	10.39 $\:\pm\:\:$2.6	8.72 $\:\pm\:\:$2.3	*F*_*2,103*_ = 11.2, *p* < 0.001
WASI Similarities	IQSIMS*	General ability-verbal	Verbal reasoning	12.30 $\:\pm\:\:$2.6	9.91 $\:\pm\:\:$2.4	8.83 $\:\pm\:\:$2.6	*F*_*2,103*_ = 16.0, *p* < 0.001
WASI Block design	IQBDS*	General ability-nonverbal	Construction	12.41 $\:\pm\:\:$2.3	9.48 $\:\pm\:\:$3.4	7.72 $\:\pm\:\:$2.5	*F*_*2,103*_ = 21.5, *p* < 0.001
WASI Matrix reasoning	IQMRS*	General ability-nonverbal	Non-verbal reasoning	12.02 $\:\pm\:\:$1.9	10.25 $\:\pm\:\:$2.5	9.22 $\:\pm\:\:$3.7	*F*_*2,103*_ = 9.7, *p* < 0.001
WRAMAL-II Total Design Recall	LLRNTDRS*	Episodic memory	Visual memory	9.14 $\:\pm\:\:$2.7	6.55 $\:\pm\:\:$2.2	6.67 $\:\pm\:\:$2.2	*F*_*2,103*_ = 14.1, *p* < 0.001
WRAMAL-II Delayed recall	LLRNDRS*	Episodic memory	Verbal memory	10.75 $\:\pm\:\:$2.4	9.20 $\:\pm\:\:$2.5	9.22 $\:\pm\:\:$3.1	*F*_*2,103*_ = 4.6, *p* = 0.012
WRAMAL-II Picture memory	WRAML	Episodic memory	Visual memory	7.82 $\:\pm\:\:$2.4	7.20 $\:\pm\:\:$2.5	7.50 $\:\pm\:\:$3.0	*F*_*2,103*_ = 0.65, *p* = 0.525
CPT-III Omission errors	CPOMT^a^*	Executive function	Inattention	21.50 $\:\pm\:\:$1.7	20.33 $\:\pm\:\:$2.8	19.44 $\:\pm\:\:$3.6	*F*_*2,103*_ = 4.7, *p* = 0.012
D-KEFS Number-Letter Seq	NUMLETSS*	Executive function	Mental tracking	10.27 $\:\pm\:\:$2.5	8.57 $\:\pm\:\:$3.0	6.39 $\:\pm\:\:$4.0	*F*_*2,103*_ = 11.2, *p* < 0.001
D-KEFS Category Switching	CATSWS*	Executive function	Verbal fluency	11.66 $\:\pm\:\:$3.5	10.20 $\:\pm\:\:$2.9	7.89 $\:\pm\:\:$3.4	*F*_*2,103*_ = 8.7, *p* < 0.001
D-KEFS Color-Word	INHSS*	Executive function	Response inhibition	11.82 $\:\pm\:\:$2.0	10.14 $\:\pm\:\:$2.8	8.28 $\:\pm\:\:$3.7	*F*_*2,103*_ = 11.6, *p* < 0.001
D-KEFS Card Sort	CORSORS*	Executive function	Problem solving	12.64 $\:\pm\:\:$2.1	10.55 $\:\pm\:\:$2.3	8.17 $\:\pm\:\:$3.3	*F*_*2,103*_ = 22.6, *p* < 0.001
D-KEFS Number Sequencing	NUMSEQS	Speed	Visuomotor speed	10.70 $\:\pm\:\:$2.6	8.98 $\:\pm\:\:$3.0	8.11 $\:\pm\:\:$4.3	*F*_*2,103*_ = 5.7, *p* = 0.004
NIH Toolbox PCPS	PCPSS *	Speed	Pattern matching	99.17 $\:\pm\:\:$22.0	95.27 $\:\pm\:\:$20.3	80.11 $\:\pm\:\:$25.0	*F*_*2,103*_ = 4.9, *p* = 0.009
D-KEFS Color Naming	COLSS	Speed	Visuo-oral speed	9.93 $\:\pm\:\:$2.7	9.66 $\:\pm\:\:$2.5	8.28 $\:\pm\:\:$2.1	*F*_*2,103*_ = 2.9, *p* = 0.062

All tests are standardized for age. WASI=Wechsler Abbreviated Intelligence Scale; WRAMAL-II=Wide Range Assessment of Memory and Learning (2nd edition); CPT-III=Continuous Performance Test (3rd edition); D-KEFS=Delis-Kaplan Executive Function System; PCPS=Pattern Completion Processing Speed. ^a^Scores are reversed so that a higher score reflects better performance. *Significantly different between groups (ANOVA corrected for multiple comparisons—see Supplemental File I).

**Table 2:** Demographic and clinical characteristics.

	Controls (*n* = 44)	Low-disadvantage JME (*n* = 44)	High-disadvantage JME (*n* = 18)
Age: mean (SD)	20.27 (3.2)	19.70 (3.9)	19.22 (3.8)
Sex (F/M)	24/20	30/14	10/8
Mother’s education*: mean (SD)	5.82 (1.7)	4.84 (1.4)	4.33 (1.8)
Age of onset (months): mean (SD)	-	166.1 (58.6)	175.0 (34.8)
Epilepsy duration (months): mean (SD)	-	69.78 (65.2)	74.75 (70.3)
Recent seizure (months): mean (SD)	-	18.47 (24.1)	10.0 (14.1)
Number of ASM (0/1/2/3)	-	1/23/16/4	0/9/9/0

*Statistically significant between groups (*p* < 0.05); SD: standard deviation; ASM: antiseizure medications.

## Graph theory analyses

### Adjacency matrices

**Fig. 1:**
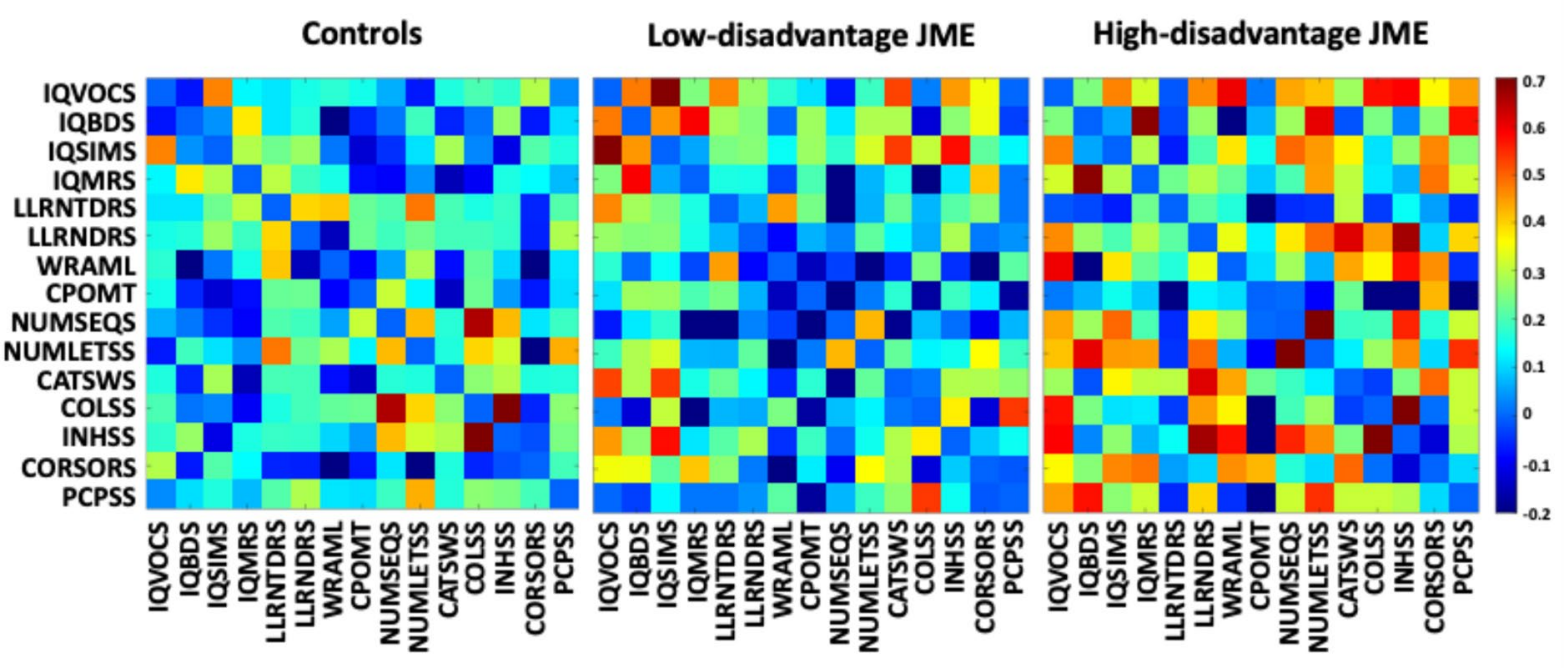
**Adjacency matrices. **Adjacency matrices of cognitive landmarks in unrelated controls (left), low-disadvantage JME (middle), and high-disadvantage JME (right). The abbreviations’ expansion can be found in Table 1.

Figure 1 shows adjacency matrices (i.e., correlation matrices) preserving both positive and negative associations between nodes (no self-correlations), and striking differences can be observed between groups, in which the high-disadvantage JME group shows higher positive correlations in general compared to both controls and low-disadvantaged (advantaged) JME. For further analyses, matrices were thresholded to preserve only positive correlations, and, indeed, high-disadvantage JME preserved the highest number of edges (i.e., links between nodes) (83%), followed by low-disadvantage JMEs (79%), and lastly by controls (77%); differences were significant (F_2,103_=13.64, *p* < 0.001) after ANOVA calculations. Therefore, most of the tests in JME were positively correlated with each other. The average strength of nodes association showed that high-disadvantage JME participants had the highest average (0.4035), followed by low-disadvantage JME (0.3634) and lastly by controls (0.3553); however, this group difference while trending was not statistically significant after ANOVA calculations (F_2,103_=2.57, *p* = 0.082. Therefore, the overall strength of associations and the actual number of positive correlations were higher in the high-disadvantage JME group, which may reflect an increased interdependence among cognitive performances, with multiple plausible theoretical explanations.

### Community structure and network hubs

**Fig. 2:**
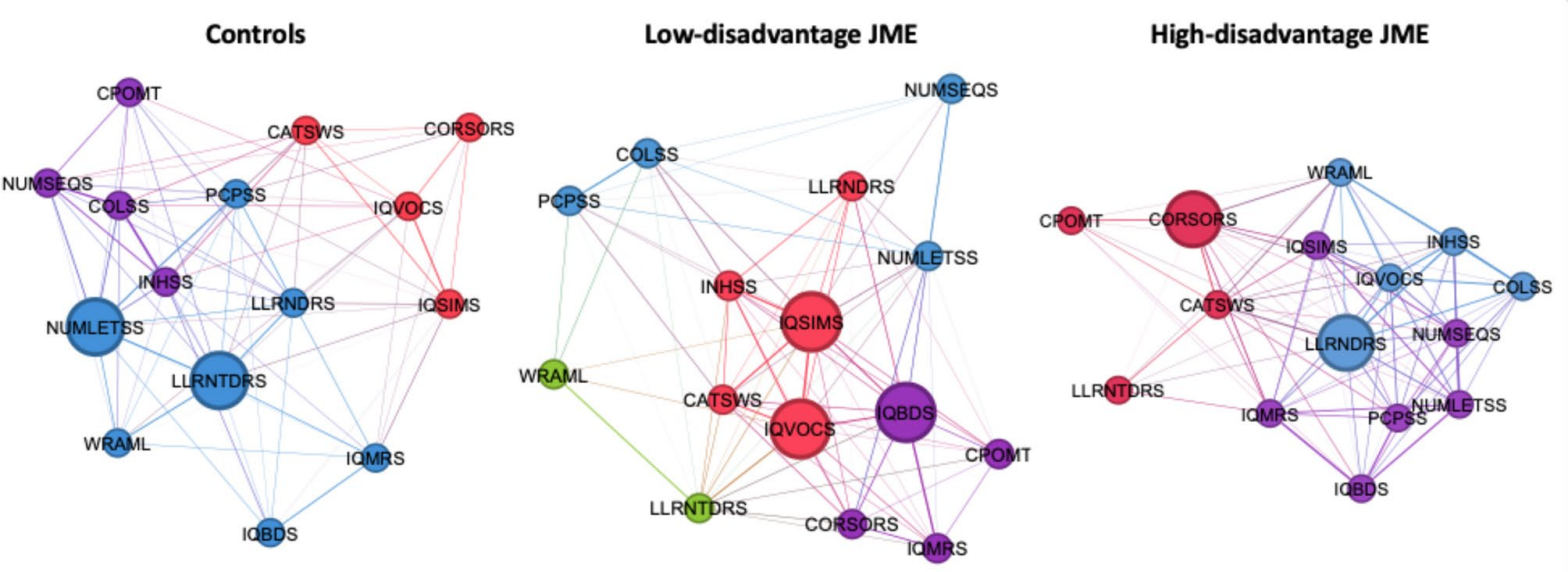
**Community structure.** Community structure of cognitive landmarks in unrelated controls (left), low-disadvantage JME (middle), and high-disadvantage JME (right). The abbreviations’ expansion can be found in Table 1. Nodes with stronger and higher number of connections are spatially closer, while those with weaker and lower number of connections are farther in space. Different colors represent different modules. Hubs –calculated using betweenness centrality– are denoted with larger circles.

In order to appreciate this further, the community structure of the cognitive landmarks of all three groups was calculated (Fig. 2). Here, it can be appreciated that the topological configuration of cognitive networks was different across groups. Controls and high-disadvantage JME presented 3 modules, while low-disadvantage JME showed 4.

Even though controls and high-disadvantage JME participants showed the same number of modules, their topological configuration was far from being similar. The largest module in controls (blue) consisted predominantly of memory (WRAML, LLRNTDRS [hub], LLRNDRS), non-verbal general ability (IQBDS, IQMRS), speed (PCPSS), and executive function (NUMLETSS [hub]). The next 2 modules had 4 nodes each: one containing verbal general ability (IQVOCS, IQSIMS), and executive function (CORSORS, CATSWS), and the other one containing executive function (CPOMT and INHSS) and speed-related nodes (NUMSEQS, COLSS).

Low-disadvantage JME showed 4 modules. The biggest contained 5 nodes (red), and it was similar in composition to one of the modules in controls (red), containing measures of verbal general ability (IQVOCS, IQSIMS) (both hubs), executive function (INHSS, CATSWS), and memory (LLRNDRS). Another module was composed of non-verbal general ability (IQBDS [hub], IQMRS), and executive function (CPOMT and CORSORS). A third module was composed of speed (COLSS, NUMSEQS, PCPSS) and executive function (NUMLETSS). There was a particularly interesting module composed of only 2 memory-related nodes (WRAML, LLRNTDRS).

In the high-disadvantage JME group, modules were less differentiated as observed in the closeness of nodes. They had 3 modules. The most populated one contained 6 nodes comprising non-verbal (IQBDS, IQMRS) and verbal general ability (IQSIMS), speed (NUMSEQS, PCPSS), and executive function (NUMLETSS). Another module contained 5 nodes comprising verbal general ability (IQVOCS), memory (WRAML, LLRNDRS [hub]), speed (COLSS), and executive function (INHSS). The third module contained 4 nodes comprising executive function (CORSORS [hub], CATSWS, CPOMT), and memory (LLRNTDRS).

**Fig. 3:**
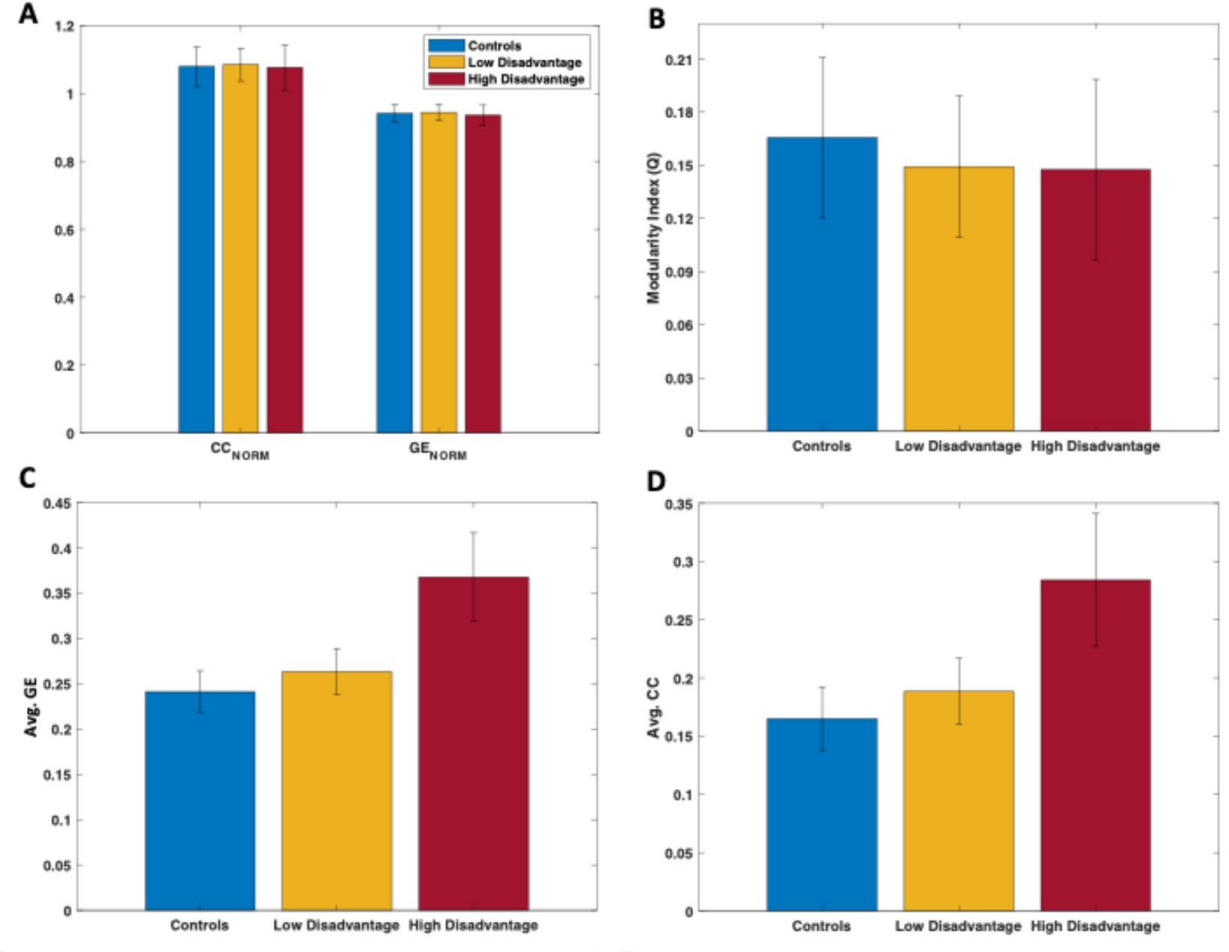
**Global graph theory measures.** (**A**) Normalized clustering coefficient (CC) and normalized global efficiency (GE), (**B**) modularity index (Q) for the cognitive networks, (**C**) Average global efficiency, and (**D**) average clustering coefficient in unrelated controls (blue), low-disadvantage JME (yellow), and high-disadvantage JME participants (red). ANOVA calculations revealed significant group differences for modularity index (F_2,103_=3.64, *p* = 0.0295; post-hoc p-values revealed statistical significance between controls and high-disadvantage JME), while trending results were found for average CC (F_2,103_=2.74, *p* = 0.0692).

Regarding the hubs, controls and high-disadvantage JME had 2, while low-disadvantage JME had 3 hubs; all related to general ability. There were no common hubs between groups. However, in controls and high-disadvantage JME, there were hubs related to memory and executive function. Altogether, it seems like the high-disadvantage JME group is failing to compensate for their cognitive deficiencies since it is showing over-integration in their cognitive network, while the low-disadvantage group seems to be relying on increased segregation of cognitive processes, suggesting a compensatory strategy to maintain performance similar to controls (Fig. 3).

### Global GT measures

Across groups, normalized clustering coefficient and global efficiency were similar (normalized CC > 1 and normalized GE < 1 for all groups), indicating comparable overall network topology. However, raw CC and GE showed a graded increase (Controls < Low-disadvantage JME < High-disadvantage JME), reflecting stronger overall associations among cognitive measures in the JME groups, although differences were not statistically significant (GE: F_2,103_=1.68, *p* = 0.1905; CC: F_2,103_=2.74, *p* = 0.0692). Modularity was highest in controls and reduced in both JME groups, with the high-disadvantage JME group showing the lowest values; statistically significant after ANOVA calculations (F_2,103_=3.64, *p* = 0.0295), with post-hoc pairwise comparisons revealing statistically significant differences between controls and high-disadvantage JME. This combination of increased raw connectivity and reduced modularity in JME suggests less differentiated cognitive networks, with the high-disadvantage JME group exhibiting the greatest reduction in network segregation. In addition, to confirm that our results are robust to network construction choices and not driven by the use of dense networks, analyses were also calculated across a range of proportional thresholds, which can be found in the supplemental Fig. 1S.

### Regression analyses

Epilepsy onset (in months), ADI scores, epilepsy duration and number of antiseizure medications were incorporated as independent variables in a regression analysis for both groups with epilepsy, with GT measures as dependent variables (average CC, average GE, Q, and average BC). For the low-disadvantage JME group, regression analysis was significant for average GE (adjusted R^2^ = 0.222, F_4,36_=3.855, *p* = 0.01), where the number of ASM was a significant predictor (*B* = 0.008, SE = 0.003, *β* = 0.399, *p* = 0.012). For the high-disadvantage JME group, the overall regression model was not significant for any of the GT measures.

## Discussion

Structural markers of disadvantage, such as the Area Deprivation Index, have been shown to have significant relationships with more problematic cognitive status, increased behavioral problems, and reduced quality of life in both youth and adults with epilepsy^5–11^. How these broad effects of disadvantage might be reflected, if at all, in detailed analyses of the nature of the test measures among themselves remains uncertain. In the context of contemporary interest in disrupted *networks* in epilepsy, we considered the cognitive metrics themselves as a network, reflected in the nature of the relationships of the test measures with each other, using the methods for “network neuropsychology”^23^.

The findings were clear and consistent—structural disadvantage was associated with disadvantageous features of the demonstrated cognitive networks. Although the normalized global efficiency and clustering coefficient did not differ across groups—with all three showing normalized CC values greater than 1 and normalized GE values below 1—indicating preserved overall network topology, the raw metrics revealed a different pattern. Both raw CC and GE increased progressively from controls to the low-disadvantaged and high-disadvantage JME groups, suggesting that the absolute strength of associations among cognitive measures was higher in the JME groups, even though the topological organization remained similar. In other words, the JME networks appeared to retain their basic architectural structure but exhibit “scaled-up” connectivity, or hyperconnectivity, with cognitive functions becoming more tightly coupled.

This interpretation is further supported by the modularity findings. The control group demonstrated the highest modularity index, consistent with a more clearly delineated community structure and greater segregation of cognitive domains. In contrast, both JME groups exhibited reduced modularity, with the high-disadvantage JME group showing the lowest values. Lower modularity, combined with stronger raw correlations, suggests reduced differentiation between cognitive subdomains, implying that functions that are normally more distinct in healthy controls become more interdependent, less distinct, and less “specialized” in JME. The slightly lower modularity in the high-disadvantage JME group may indicate an additional erosion of network specialization associated with socioeconomic disadvantage. Taken together, these results point to a pattern in which network topology is preserved, but the balance between segregation and integration shifts, with JME—particularly under higher disadvantage—showing stronger, less modular, and more interlocked cognitive networks.

ADI likely captures cumulative environmental exposures associated with disadvantaged neighborhoods, including reduced educational resources, poorer nutrition, and limited cognitive enrichment, which may contribute to differences in cognitive organization observed across groups. These findings bear a relationship to other features of JME, which, as a disorder with onset predominantly during adolescence, has the potential to disrupt normal neurodevelopmental processes. In fact, we have reported the relationship between JME and slowed cortical and thalamic maturation characterized by increased cortical thickness and/or volume in premotor as well as broader frontal regions, along with disruption of typical motor thalamic-premotor frontal associations^33^. This arguably “dysmature” pattern of brain development is reflected in the cognitive networks reported here. Both JME groups show less differentiation of cognitive networks, a typical developmental process that can be observed in the controls, which is exacerbated in the JME group exposed to greater disadvantage.

### Study limitations and future directions

This study has several limitations that should be considered when interpreting the findings. First, the sample sizes across groups were unequal, with one group including 18 participants (high deprivation JME) compared to 44 in the other two groups (low deprivation JME and controls). This imbalance may have reduced statistical power in the smallest group, particularly in the regression analyses where no significant predictors were identified, and may have served to increase susceptibility to sampling variability. Second, neuropsychological tests, even when subjected to advanced analytical methods, provide only static snapshots of cognition and cannot fully capture the dynamic, context-dependent interactions among cognitive abilities that occur in daily life. Thus, while our findings offer novel insight into the static relationships among cognitive domains—and how these differ in epilepsy compared to controls—these patterns should be interpreted with caution, as their real-world expression has yet to be empirically demonstrated. Without repeated assessments it is difficult to infer stability in cognitive network organization which should be a direction for future research. Third, given the limited representation of high-ADI individuals in the control group (*n* = 2), we were unable to examine ADI-related effects within controls; thus, the extent to which differences between low- and high-deprivation groups are specific to JME remains uncertain. Together, these methodological constraints may affect the generalizability of the results and highlight the need for replication in larger and more balanced samples. Fourth, while we did not detect significant associations between network typology and clinical seizure correlates such as seizure frequency or time since last seizure, the known inaccuracy of patient-provided seizure counts compared to direct ictal recordings remains a major limitation in this area of research. These relationships need to be examined in more controlled settings (e.g., in an epilepsy monitoring unit) or with research-friendly technology (wearable EEG caps with continuous recordings). Finally, in this investigation a number of properties were computed with the aforementioned modest sample size. As such, the stability of the network topology estimates and centrality indices remain to be determined. Future work with larger sample sizes should be undertaken to derive stronger stability analyses with replication to ensure the findings reported here represent genuine alterations in cognitive network organization.

Ferguson^23^ identified alternative approaches to network neuropsychology including classical cognitive neuropsychology grounded in a lesion-based approach, use of latent variable models that infer underlying cognitive constructs, and information-processing frameworks that emphasize a modular or hierarchical cognitive organization. Our view is that associative-based network analyses are applicable to the task undertaken here, are very underexplored in epilepsy, offer new insights into the results from the classic neuropsychological tests used in everyday practice, and align well with the latest thinking of epilepsy as a network disorder. Nevertheless, other approaches should be considered in the future.

## Conclusions

In this exploratory investigation using high-dimensional network modelling to interrogate associations between variations in cognitive networks as a function of the degree of experienced neighborhood deprivation, variations in the topological structure of the cognitive networks are found to covary with deprivation.

## Supplementary Information

Below is the link to the electronic supplementary material.

Supplementary Material 1

## Data Availability

The datasets generated during and/or analyzed during the current study are available from the corresponding author on reasonable request.
