# Supplementary material for: The network neuropsychology of neighborhood deprivation in juvenile myoclonic epilepsy
Source: Sci Rep. 2026 Apr 16;16:17662. doi: 10.1038/s41598-026-46473-2 (PMC13243532; doi:10.1038/s41598-026-46473-2)
Supplement: Supplementary file 1 — Supplementary Material 1 [file 41598_2026_46473_MOESM1_ESM.pdf]

Supplemental File

I. General neuropsychological status

The same tests used for the graph theory analysis were directly compared between groups using multivariate analysis of variance (MANOVA). Box's M test was significant ($p < 0.001$), indicating violations of covariance equality. Given moderate group-size imbalance, Pillai's Trace — the most robust statistic — was used for interpreting multivariate effects, which was significant (Pillai's Trace = 0.655, $F_{30,180} = 2.92$, $p < 0.001$). Because the overall MANOVA was significant, follow-up univariate ANOVAs were examined for each dependent variable. Significant group effects emerged for word knowledge (IQVOCS), perceptual construction (IQBDS), verbal reasoning (IQSIMS), non-verbal reasoning (IQMRS), verbal list learning and delayed recall (LLRNTDRS, LLRNDRS), inattention (CPOMT), working memory (NUMLETSS), psychomotor speed (NUMSEQS), verbal working memory (CATSWS), response inhibition (INHSS), novel problem solving (CORSORS), and speeded pattern matching (PCPSS). For the remaining dependent variables, including word reading (WRAML) and speeded color naming (COLSS), the group effect was not statistically significant (all *ps* > 0.05).

Post hoc pairwise comparisons (Bonferroni-adjusted) demonstrated that the pattern of differences across groups varied by measure. In general, group differences were significant between controls and each JME subgroup but not between each JME subgroup (IQVOCS, IQBDS, IQSIMS, IQMRS, LLRNTDRS, NUMSEQS, and INHSS). For NUMLETSS and CORSORS, all groups were significantly different from each other. For CATSWS and PCPSS, differences were between controls and high ADI JME, and between both JME subgroups. CPOMT only showed significant post-hoc differences between controls and high ADI JME.

II. Sensitivity analyses: GT measures calculations for a range of proportional thresholds

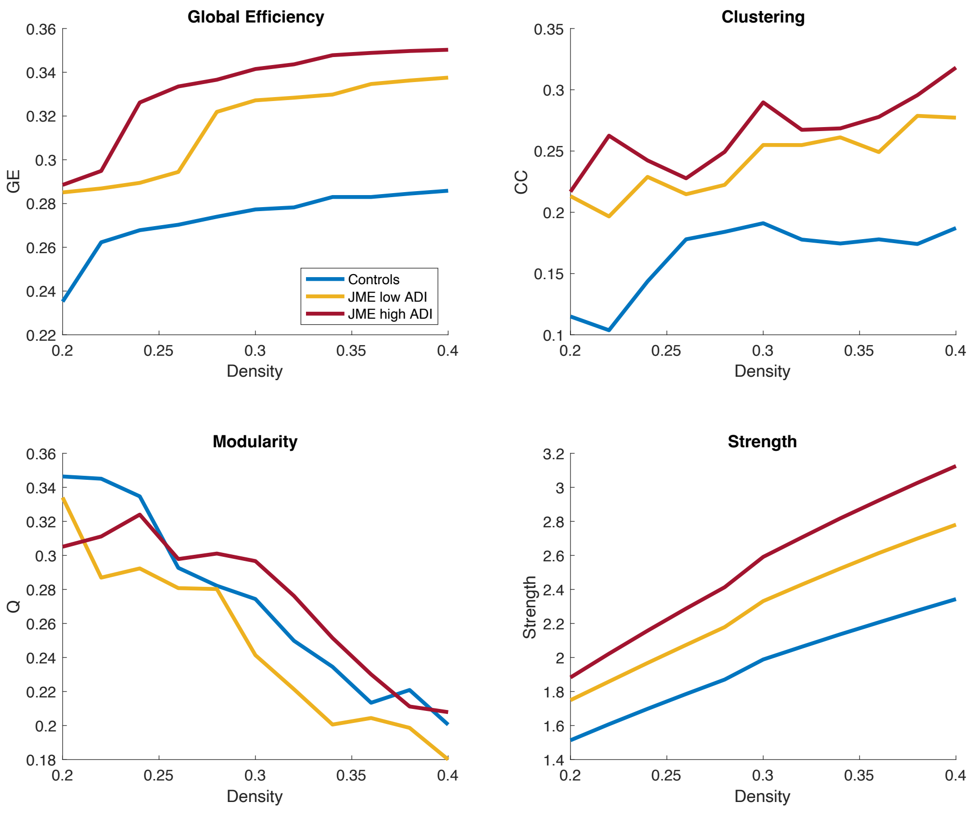

Figure 1S: Global efficiency, average clustering coefficient, modularity index, and average strength for controls (blue), low-disadvantage JME (yellow), and high-disadvantage JME (red) for a range of proportional thresholds.
